# *Cystin* genetic variants cause autosomal recessive polycystic kidney disease associated with altered *Myc* expression

**DOI:** 10.1038/s41598-021-97046-4

**Published:** 2021-09-14

**Authors:** Chaozhe Yang, Naoe Harafuji, Amber K. O’Connor, Robert A. Kesterson, Jacob A. Watts, Amar J. Majmundar, Daniela A. Braun, Monkol Lek, Kristen M. Laricchia, Hanan M. Fathy, Shrikant Mane, Shirlee Shril, Friedhelm Hildebrandt, Lisa M. Guay-Woodford

**Affiliations:** 1 Center for Translational Research, Children’s National Research Institute, 111 Michigan Ave NW, Washington, DC 20010 USA; 2 Department of Genetics, University of Alabama at Birmingham, Birmingham, AL 35294 USA; 3 Department of Medicine, Boston Children’s Hospital, Harvard Medical School, Boston, MA 02115 USA; 4 Program in Medical and Population Genetics, Broad Institute of MIT and Harvard, Cambridge, MA USA; 5 Alexandria Faculty of Medicine, University of Alexandria, Alexandria, Egypt; 6 Department of Genetics, Yale University School of Medicine, New Haven, CT USA; 7 Yale Center for Mendelian Genomics, Yale University School of Medicine, New Haven, CT USA

**Keywords:** Polycystic kidney disease, Disease genetics, Experimental models of disease

## Abstract

Mutation of the *Cys1* gene underlies the renal cystic disease in the *Cys1*^*cpk/cpk*^ (*cpk*) mouse that phenocopies human autosomal recessive polycystic kidney disease (ARPKD). Cystin, the protein product of *Cys1*, is expressed in the primary apical cilia of renal ductal epithelial cells. In previous studies, we showed that cystin regulates *Myc* expression via interaction with the tumor suppressor, necdin. Here, we demonstrate rescue of the *cpk* renal phenotype by kidney-specific expression of a cystin-GFP fusion protein encoded by a transgene integrated into the *Rosa26* locus. In addition, we show that expression of the cystin-GFP fusion protein in collecting duct cells down-regulates expression of *Myc* in *cpk* kidneys. Finally, we report the first human patient with an ARPKD phenotype due to homozygosity for a deleterious splicing variant in *CYS1*. These findings suggest that mutations in *Cys1*/*CYS1* cause an ARPKD phenotype in mouse and human, respectively, and that the renal cystic phenotype in the mouse is driven by overexpression of the *Myc* proto-oncogene.

## Introduction

Autosomal recessive polycystic kidney disease (ARPKD; MIM 263,200) affects 1:26,500 live births^1^. Cohort studies indicate that 80% or more of patients with typical ARPKD have variants in the Polycystic Kidney and Hepatic Disease 1 (*PKHD1*) gene^2–6^. Variants in the *DZIP1L* gene account for less than 1% of affected patients^7^, while variants in other hepato-renal fibrocystic disease (HRFD) genes, eg. *HNF1B*, *PKD1*, *NPHP2*, *NPHP3*, and *NPHP13,* can phenocopy ARPKD^8^. For reasons yet to be explained, mice with targeted disruption of *Pkhd1* exhibit little or no kidney disease^9–16^. In the absence of a *Pkhd1* mutant mouse model that accurately recapitulates the human disease phenotype, the *cpk* mouse carrying a spontaneous truncating mutation in *Cys1* has been the most widely studied mouse model of ARPKD^17,18^. Cystin, the *Cys1* gene product, is a 145-amino acid cilia-associated protein that is expressed in mouse embryonic kidney and liver ductal epithelium^19^. Disruption of cystin function results in elevated *Myc* expression in collecting duct epithelial cells^20–23^ and increased cell proliferation^20,24^. In previous work, we have demonstrated that in renal collecting duct epithelia, cystin physically interacts with necdin in a regulatory complex that modulates *Myc* expression^25^.

Cystin deficiency-associated disruption of ciliary signaling and/or overexpression of *Myc* is associated with aberrant SMAD3 phosphorylation^26^, overexpression of *Fos* and *Kras* proto-oncogenes^20–22^, elevated levels of growth factors^27^, aberrant localization and abundance of the epidermal growth factor receptor (EGFR) on the apical surface of collecting duct cells^28^ and altered levels of basement membrane components^29–31^ and epithelial cell adhesion molecules^32,33^. Until now, the relevance of these effects of cystin deficiency for human disease was unclear in the absence of ARPKD patients with variants in human *CYS1*. Here we present the first case of human ARPKD due to homozygosity for a *CYS1* variant, in this case predicted to cause defective splicing. We also show that complementation of defective *Cys1* in *cpk* mouse kidneys rescues both *Myc* overexpression and the collecting duct cyst phenotype. These studies suggest that up-regulation of *Myc* expression in vivo may play a central role in the pathogenesis of mouse recessive polycystic kidney disease (PKD), with important implications for human ARPKD.

## Results

### Phenotypic rescue of *cpk* mice by kidney-specific expression of a cystin-GFP fusion protein

**Figure 1:**
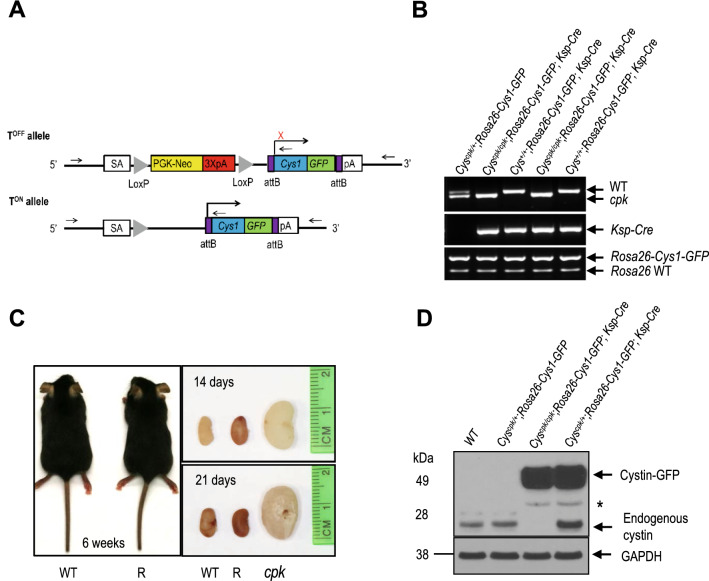
Rescue of *cpk* mouse phenotype by kidney-specific expression of Cys1-GFP. (**A**) Schematic diagram showing the *Cys1-GFP* transgene knock-in at the *Rosa26* locus (*Rosa26-Cys1-GFP* allele) before (T^OFF^) and after (T^ON^) deletion of a PGK Neo cassette (yellow rectangle) flanked by LoxP sites (gray triangles). In the T^OFF^ configuration, expression of *Cys1-GFP* is prevented by the PGK Neo cassette. In cells expressing a *Ksp-Cre* transgene, Cre-mediated recombination deletes PGK Neo and *Cys1-GFP* is expressed (T^ON^). SA: splice acceptor, PGK-Neo: Phosphoglycerate kinase promoter driving a neomycin resistance gene followed by 3 polyA signals (3XpA, red rectangle). The purple boxes flanking *Cys1-GFP* are attB sites. (**B**) PCR-based genotyping of *Rosa26-Cys1-GFP*, *Ksp-Cre* and *Cys1* alleles in mice of the indicated genotypes. (**C**) *Cys1-GFP* rescue of gross phenotypes in *cpk* mice. Six-week-old wild-type (WT) and *Cys1*^*cpk/cpk*^; *Rosa26-Cys1-GFP; Ksp-Cre/* + rescued (R) mice are of equivalent size. Examination of kidneys from WT, R and *cpk* mice at 14 and 21 days of age show equivalent sizes of R and WT kidneys, with both markedly smaller than *cpk* kidneys. (**D**) Western blot analysis of total kidney protein from 6-week-old mice of the indicated genotypes. Mouse cystin is 145 amino acids long but migrates aberrantly at ~ 25 kDa on SDS-PAGE. Cystin-GFP (arrow, ~ 50 kDa) and endogenous cystin (arrowhead, ~ 25 kDa) were detected using polyclonal rabbit anti-cystin antibody, as previously described^19^. GAPDH served as an internal protein loading and transfer control. The asterisk indicates non-specific bands.

We generated a conditional expression *Cys1* transgenic *Cys1*^*cpk/cpk*^* (cpk)* mouse line carrying a *Cys1-GFP* transgene knock-in at the *Rosa26* locus. In these mice, *Cys1-GFP* transgene expression is precluded by the presence of a loxP-flanked termination sequence consisting of a PGK-Neo cassette (Fig. 1A, T^OFF^ allele). The *Cys1*-GFP transgene is expressed by the ROSA26 promoter only after *Cre*-mediated deletion of the loxP-flanked PGK-Neo cassette (Fig. 1A, T^ON^ allele). We crossed *Rosa26-Cys1-GFP* mice with *Cys1*^*cpk/*+^ mice to generate *Cys1*^*cpk/*+^; *Rosa26-Cys1-GFP* mice, which were then crossed with *Ksp-Cre* transgenic mice^34^ to generate *Cys1*^*cpk/*+^; *Rosa26-Cys1-GFP*; *Ksp-Cre* progeny. In these mice, *Cre* expression, controlled by the *Ksp*-cadherin regulatory elements, occurs exclusively in the developing distal renal tubular epithelium and the genitourinary tract^35^, resulting in high level expression of the cystin-GFP fusion protein in the collecting ducts and loops of Henle and low or no expression in the proximal tubules. Finally, the rescue experiments were carried out by crossing *Cys1*^*cpk/*+^; *Rosa26-Cys1-GFP*; *Ksp-Cre* mice with *Cys1*^*cpk/*+^ mice. Genotype-confirmed *Cys1*^*cpk/cpk*^; *Rosa26-Cys1-GFP*; *Ksp-Cr*e experimental “rescue” (R) mice were compared to their *Cys1*^+*/*+^; *Rosa26-Cys1-GFP*; *Ksp-Cre* control (C) littermates (Fig. 1B). While *cpk* mice are characteristically smaller than wild-type littermates and die by 21 days of age^36^, no differences were observed between R mice and their littermate controls with respect to body size (Fig. 1C, left panel) or viability/lifespan (R mice were routinely euthanized at 12 months of age, as were normal C mice). Kidney sizes at postnatal days 14 and 21 were not significantly different in R and wild-type (WT) mice (Fig. 1C, right panel), while age-matched *Cys1*^*cpk/cpk*^ (*cpk*) mice exhibited the characteristic cystic kidney phenotype. These results indicate that the *cpk* phenotype was rescued by kidney-specific expression of cystin-GFP.

### Expression of cystin-GFP fusion protein in the kidneys of rescued *cpk* mice

We examined the expression of the cystin-GFP fusion protein in the kidneys of R mice. Endogenous cystin was detectable in the kidneys of both WT and C mice and absent from the kidneys of R mice (Fig. 1D). The cystin-GFP fusion protein of ~ 50 kDa was detected in R and C mice (Fig. 1D, lanes 3 and 4). These results demonstrate that cystin-GFP expression was associated with Cre-mediated excision of the PGK-Neo cassette.

**Figure 2:**
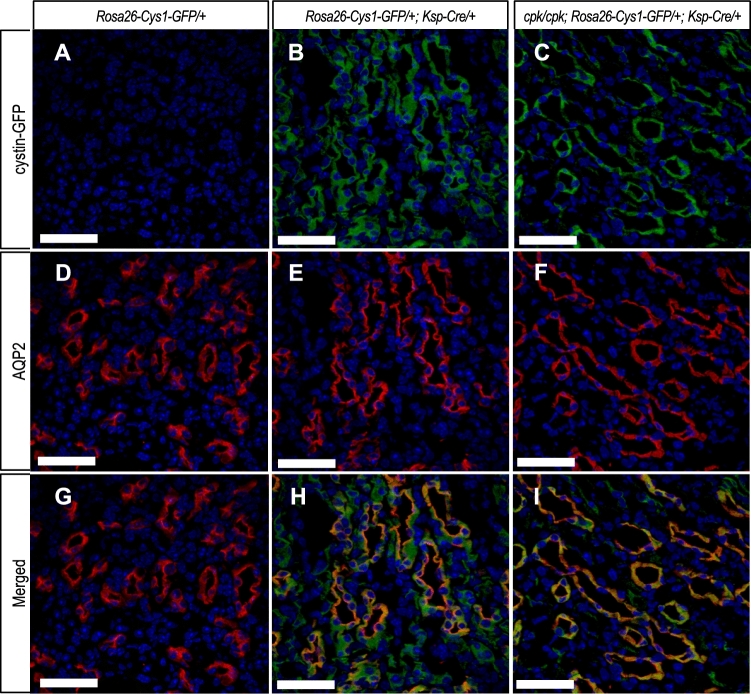
Cystin-GFP fusion protein expression in renal collecting duct. Immunohistochemical detection of cystin-GFP fusion protein (green, **A**–**C**), AQP2 (red, **D**–**F**) and merged (**G**–**I**) in kidney tissues from 6-week-old mice of the indicated genotypes. The *Rosa26-Cys1-GFP/*+ mice (**A**, **D**, **G**) do not express cystin-GFP fusion protein due to the absence of the *Ksp-Cre* transgene. The *Rosa26-Cys1-GFP/*+; *Ksp-Cre/*+ mice (**B**, **E**, **H**) have a wild-type *Cys1* gene and express cystin-GFP fusion protein in Cre-positive cells. The *cpk/cpk; Rosa26-Cus1-GFP/*+; *Ksp-Cre/*+ rescue mice (**C**, **F**, **I**) express cystin-GFP fusion protein in Cre-positive cells. Cell nuclei are stained with DAPI (blue). Scale bars equal 50 µm. Images are representative of tissue sections from 3 animals.

Immunofluorescence staining with antibodies against GFP (Fig. 2A–C) and aquaporin-2 (AQP2; Fig. 2D–F) was used to examine cystin-GFP expression in nephron segments of kidneys from R and C mice. AQP2 is expressed primarily on apical cell membranes of collecting duct cells^37,38^. The cystin-GFP fusion protein was detected in AQP2-positive collecting ducts of R mice (Fig. 2C, I) and C mice (Fig. 2B, H), while cystin-GFP was absent in *Rosa26-Cys1-GFP* mice, that lack a *Ksp-Cre* transgene (Fig. 2A, G). Co-localization of AQP2 and cystin-GFP demonstrated cystin-GFP fusion protein expression in the collecting duct cells.

### Histological evaluation of cystogenesis in rescued *cpk* mice

**Figure 3:**
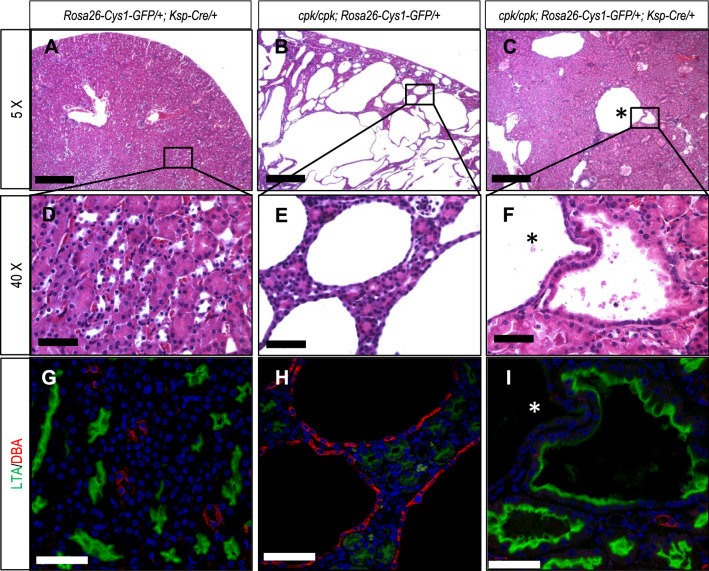
Overall attenuation of renal cystic phenotype in *cpk* rescue mice, and sporadic proximal tubule cyst formation. Formalin fixed, paraffin embedded 5 µm sections of kidney tissues from 6-week-old mice of the indicated genotypes were stained with H&E and examined by light microscopy (**A**–**F**) or stained with lectins LTA (green, proximal tubules) or DBA (red, distal tubules) and examined by immunofluorescence microscopy (**G**, **H**, **I**). Panels (**A**–**C**) are ×5 magnification with scale bars equal to 200 µm. Boxed areas are shown at ×40 magnification in corresponding panels (**D**–**F**) with scale bars equal to 50 µm. Lectin staining was performed on serial sections corresponding to H&E stained samples. Cell nuclei were stained with DAPI. Scale bars in panels (**G**–**I**) are equal to 50 µm. The asterisk (*) identifies a large cyst that was not stained by either LTA or DBA. Images are representative of tissue sections from 3 animals.

The gross evaluation of kidneys from R mice suggested that the renal histology would be normal. Histological evaluation showed that, while the majority of the nephrons in R kidneys appeared to have normal dimensions, occasional cystic structures were present (Fig. 3A–F). Using DBA and LTA lectins, markers of distal and proximal tubules, respectively^39,40^, we observed (Fig. 3G–I) that the cystic structures stained with LTA (Fig. 3I). These findings suggest that while expression of cystin-GFP in collecting ducts markedly attenuated the *cpk* renal phenotype, sporadic cyst formation did occur in proximal tubular segments of these kidneys (Fig. 3I). We also showed (Supplementary Fig. S1) that cystin-GFP was expressed in R kidneys throughout the cortical and medullary regions, but was not expressed in glomeruli. These examinations also showed that sporadic cysts formed predominantly along the cortico-medullary junction, corresponding to proximal tubule involvement.

### Expression of *Myc* in rescued *cpk* mice

**Figure 4:**
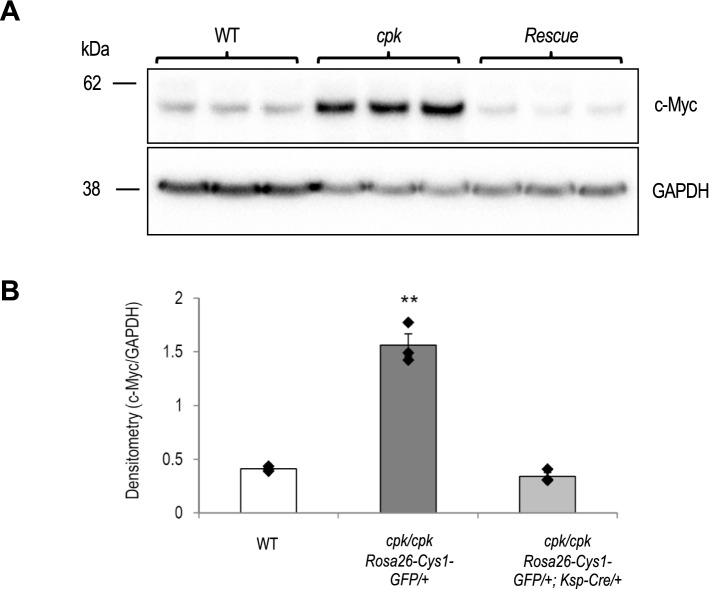
Decreased c-MYC expression in kidneys of *cpk* rescue mice. Immunoblot analysis of c-MYC protein expression in kidneys from wild-type (WT), *cpk/cpk*; *Rosa26-Cys1-GFP/*+ (*cpk*) and *cpk/cpk*; *Rosa26-Cys1-GFP/*+; *Ksp-Cre/*+ (Rescue) mice. (**A**) Total kidney lysates were immunoblotted using anti-c-MYC and anti-GAPDH antibodies. (**B**) c-MYC band intensity was normalized with GAPDH. Data represents mean ± S.E; Y-axis values indicate c-MYC/GAPDH band intensity ratio; **P < 0.01, versus others, ANOVA, n = 3 each group.

*Myc* overexpression in *cpk* kidneys is well-documented^20–23^. In previous work we demonstrated that cystin physically interacts with the DNA-binding protein necdin in a regulatory complex that binds to the *Myc* P1 promoter^25^. Necdin enhances *Myc* promoter activity and cystin antagonizes this effect. In a previous report, we proposed that *Myc* up-regulation in *cpk* kidneys results directly from disruption of the cystin-necdin interaction. In the current study, we examined the relative abundance of c-MYC protein in the kidneys of 14-day old R mice as compared to *cpk* mice. Quantitative immunoblotting revealed comparable levels of c-MYC in WT and R kidneys that were markedly lower than in kidneys of *cpk* mice (Fig. 4). These results demonstrate that transgene rescue of the cystic kidney phenotype in *cpk* mice is associated with down-regulation of c-MYC protein expression, suggesting a central role for *Myc* overexpression in renal cystogenesis in this mouse model.

Cystin regulation of *Myc* gene expression occurs via cystin-necdin mediated repression of *Myc* transcription^25^. We therefore used qRT-PCR to assess relative *Myc* mRNA levels in kidney samples from 14-day-old B6 WT, *cpk* and R mice (Supplementary Fig. S2). We observed that, consistent with Myc western blotting results, *Myc* mRNA levels were upregulated in *cpk/cpk* cystic kidney but normal in kidneys from R mice. These findings further support a central role of *Myc* gene overexpression in *cpk* renal cystogenesis.

### Recessive *CYS1* variant in an individual with childhood cystic kidney disease and liver fibrosis

While the *cpk* mouse phenotype recapitulates important clinical features of ARPKD, to date no human cases of the disease have been linked to variants in the *CYS1* gene. We evaluated a case of a 5-year-old male child, subject B783, born from a consanguineous union, who presented with polyuria, polydipsia and poor growth. Renal ultrasonography showed multiple medullary and cortical cysts concerning for polycystic kidney disease. Renal function was mildly reduced (creatinine 0.6 mg/dL) for age.

**Figure 5:**
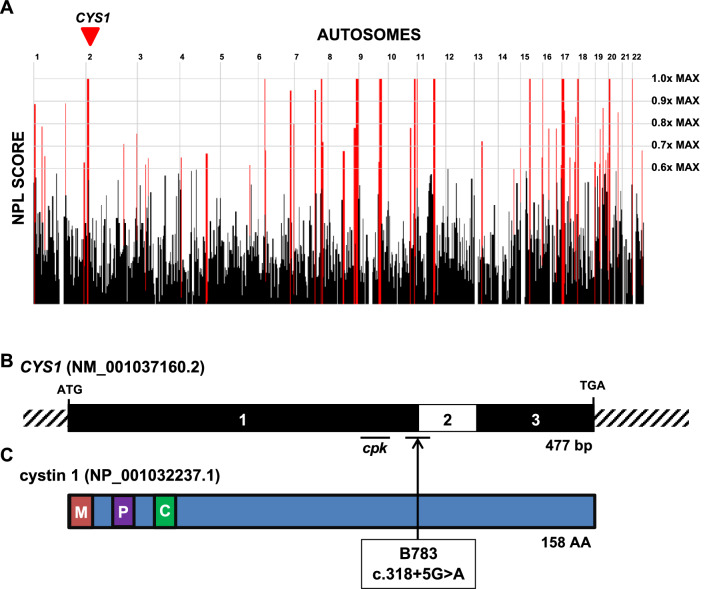
A recessive variant in *CYS1* associated with polycystic kidney disease and liver fibrosis. (**A**) Genome-wide homozygosity mapping of B783 identifies homozygous peak regions. *CYS1* is located within a peak region of chromosome 2 (arrowhead). (**B**) Exon structure of *CYS1* cDNA. The locations of start codon, stop codons, and affected splice site (boundary exon 1–2) are indicated. The region of exon 1 deleted in the *cpk* mouse is also indicated. (**C**) Protein domain structure of cystin-1. A myristoylation site (M), polybasic region (P) and AxEGG motif required for targeting cystin-1 to the cilium (C) are indicated. Arrow indicates the boundary between protein regions encoded by exons 1 and 2. Abbreviations: AA, amino acid; bp, base pairs; C, cilium trafficking domain; M, myristoylation site; P, polybasic region.

To identify a monogenic cause of disease, we performed trio exome sequencing (TRIO-ES) on DNA samples obtained from the proband and parents. Homozygosity mapping from exome variant data for B783 demonstrated 108 Mbp of homozygosity by descent (Fig. 5A), suggesting the parents are approximately fourth degree relatives. Based on this mapping, we hypothesized that a biallelic gene variant residing within a homozygous peak region underlies proband renal disease. To identify the most probable disease-causing variant, we used the following criteria for exome variant filtering^41–44^: (1) exclusion of all variants that did not change the amino-acid sequence or affected canonical splice sites (defined as ± 6 nucleotides surrounding the exon–intron boundary), (2) exclusion of variants reported in the homozygous state or with a minor allele frequency greater than 0.1% in a control cohort (ExAC and gnomAD genome databases), (3) inclusion of homozygous bi-allelic variants with appropriate parental segregation consistent with our above hypothesis, and (4) assessment of variants for deleteriousness based on in silico prediction of their impact on protein structure and/or splice site function. This approach failed to identify a strong candidate variant. We, therefore, performed trio genome sequencing (TRIO-GS), which provides increased coverage of non-coding regions.

Using the same filtering approach as described above for TRIO-ES, we identified a homozygous splice-site variant (c.318 + 5G > A) in the exon 1 donor site in the *CYS1* gene (Fig. 5B, C). Conceivably, this variant was not detected by TRIO-ES because *CYS1* exon 1 was under-represented due to its high GC-content, which can result in low capture efficiency during the generation of sequence libraries for WES (and see Discussion)^45^. Consistent with our hypothesis, this variant resides within a region of homozygosity of descent (Fig. 5A). It is deleterious based on the following criteria: (i) the splice variant is extremely rare, as it is absent in the gnomAD database (gnomAD, version 2.1.1), and (ii) the variant was predicted to have deleterious effect on splicing using four independent in silico prediction tools (Table 1). Importantly, in both TRIO-ES and TRIO-GS, we did not identify causative variants in 100 cystic kidney disease genes including *PKHD1* or *DZIP1L*. Allele-specific PCR confirmed the variant was homozygous in the affected child and heterozygous in the parents (Supplementary Fig. S3).

**Table 1:** Recessive variant in *CYS1* in one family with cystic kidney disease.

Family	Nucleotide change	Amino acid change	Exon (Zyg, Seg)	In silico Severity Scores	gnomAD (H/h/T)	Sex	Ethnic origin	PC	Clinical phenotype
B783	c.318 + 5G > A	Splice	1 (Hom, B)	*CADD* 17.06^a^ *HSF* 13.17^b^ *MaxEnt* 62.8%^c^ *NNS* 71.3%^c^	0/0/ ~ 251,000	M	Egypt	Y	Initial Onset: 5 years, polyuria, polydipsia Serum Studies: Cr 0.6 mg/dL RUS: bilateral cortical and medullary cysts Extra-renal: Mild congenital hepatic fibrosis

B, both parents appropriately heterozygous; CADD, Combined Annotation-Dependent Depletion prediction score; Cr, serum creatinine; gnomAD, Genome Aggregation database; H, homozygotes in gnomAD; h, heterozygous alleles in gnomAD; Hom, homozygous zygosity; HSF, HSF splice prediction score; M, male; MaxEnt, MaxEnt splice prediction score; NNS, NNSPLICE splice-site variant prediction score; PC, parental consanguinity; RUS, renal ultrasound; Seg, segregation; T, total alleles in gnomAD; Y, yes; Zyg, zygosity.

^a^CADD scores between 10 and 20 are in the 1–10% most deleterious substitutions possible in human genome in terms of predicted effect on the gene product.

^b^Predicts wildtype site broken by this change.

^c^Percentage reflects expected reduction in splicing at this donor site as consequence of change.

Based upon these findings, the clinical data of subject B783 was further queried. Prenatal ultrasonography showed polyhydramnios and echogenic, but normal sized, kidneys. B783 did not develop respiratory distress after birth and had no subsequent pulmonary problems. Similarly to the established phenotype observed in *cpk* mice^46–48^ (on some genetic backgrounds), he exhibited mild congenital liver fibrosis (Fig. 5B) but did not have abnormalities in liver function tests. Moreover, his renal function worsened. By age 11 years, he had progressed to end-stage kidney disease and was on dialysis. Overall, the renal-hepatic syndromic features of subject B783 further support that the homozygous *CYS1* variant c.318 + 5G > A is likely pathogenic.

Based on this unique case, we analyzed ES data from 521 individuals with pediatric onset cystic kidney disease to identify additional families with deleterious variants in the *CYS1* locus but did not identify any additional cases. Our findings strongly suggest the first identification of a causative variant in *CYS1* in a human patient with an ARPKD phenotype.

## Minigene assay confirms an effect of *CYS1* variant c.318 + 5G > A on splicing

**Figure 6:**
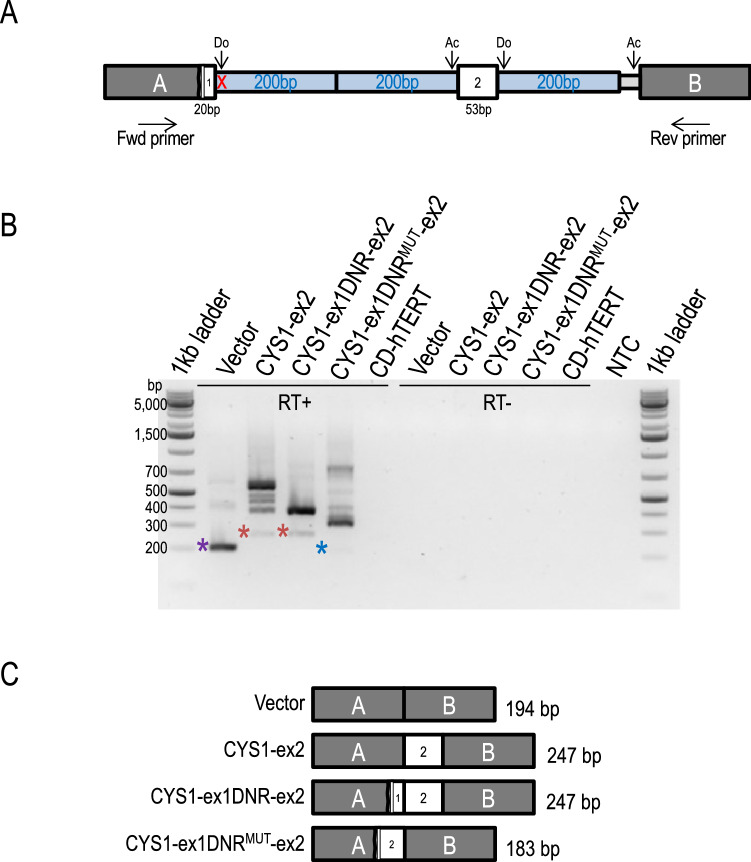
*CYS1* variant c.318 + 5G > A caused abnormal splicing of RNA expressed from a transfected minigene in human renal cells. (**A**) Experimental minigene splicing constructs were generated by cloning the following *CYS1* sequences between vector exons A and B: 20 bp of *CYS1* exon 1 3’ sequence including either the WT or the c.318 + 5G > A patient-derived variant splice donor site; then 200 bp of adjacent intron1 sequence; then *CYS1* sequence consisting of the entire *CYS1* exon 2 (53 bp) flanked by 200 bp of adjacent *CYS1* intronic sequences. Minigene constructs were transfected into human renal collecting duct (CD-hTERT) cells^85^. Normal splice donor (Do) and acceptor (Ac) sites are indicated by arrows. Forward (Fwd) and reverse (Rev) primers corresponding to sequences in vector exons A and B, respectively, were used in RT-PCR. The position of the c.318 + 5G > A patient-derived variant is indicated with a red X. (**B**) RT-PCR reaction products (using Fwd & Rev primer pair) from CD-hTERT RNA following transfection with the indicated plasmids. Amplicons were resolved by agarose gel electrophoresis. RT+, addition of reverse transcriptase; RT-, negative control reactions lacking reverse transcriptase. CD-hTERT indicates template RNA from untransfected cells used as a negative control. NTC, no template control. The vector positive control generated an amplicon of approximately 200 bp, as expected (purple asterisk). CYS1-ex2 and CYS1-ex1DNR-ex2 generated amplicons of the expected 250 bp size (red asterisks). In contrast, the c.318 + 5G > A variant showed loss of the 250 bp amplicon and the appearance of a novel, shorter amplicon (blue asterisk). The identities of the bands marked with asterisks were confirmed by cloning of excised DNA and subsequent restriction and sequence analysis (Supplementary Data). (**C**) RT-PCR amplicon composition and size (bp) corresponding to splicing products generated from transfected minigene constructs amplified using the Fwd and Rev primer pair (shown in **A**). Outcomes for the variant construct, assuming an effect of the *CYS1* variant on splicing, could not be predicted a priori. The composition of the splicing product shown here is from the actual sequence of the cloned amplicon (see Supplementary Data). Sequences of cloned splice product amplicons from all other constructs corresponded precisely to anticipated outcomes.

We were unable to obtain samples of patient-derived mRNA needed to directly ascertain effects of the variant on *CYS1* gene splicing. As an alternative strategy, we performed minigene-based assays^49^ that allowed comparison of RNA splicing products derived from *CYS1* WT to those from the c.318 + 5G > A allele minigene constructs transfected into human renal collecting duct cells. As illustrated in Fig. 6A, *CYS1* DNA sequences were cloned between vector pSpliceExpress exons A and B such that: 20 bp of *CYS1* exon 1 3’ sequence, including either the WT (construct CYS1-ex1DNR-ex2, in which “DNR” denotes “WT donor”) or the c.318 + 5G > A patient-derived variant splice donor site (CYS1-ex1DNR^MUT^-ex2), replaced 3’ exon A vector sequences, followed by 200 bp of adjacent *CYS1* intron 1 sequence, followed by *CYS1* sequence consisting of the entire *CYS1* exon 2 (53 bp) flanked on either side by 200 bp of adjacent *CYS1* intronic sequences. A positive control construct was also generated (CYS1-ex2), consisting of *CYS1* exon 2, flanked on either side by 200 bp of native *CYS1* intron sequences, ligated between vector exons A and B. The constructs were transfected into human renal collecting duct cells, RT-PCR (using the Fwd & Rev primer pair) was performed on extracted mRNA, amplicons were resolved by electrophoresis through agarose gels and analyzed by restriction digestion and DNA sequencing to resolve the composition of relevant mRNA splicing products derived from the transfected plasmids (Fig. 6B). RT-PCR product sizes from parent vector, CYS1-ex2 positive control, and CYS1-ex1DNR-ex2 containing WT donor sequence yielded amplicons equivalent in size to the expected splice products, verified by subcloning and DNA digestion and sequencing (Fig. 6C and Supplementary Data). Higher molecular weight amplicons correspond in size to anticipated unspliced or intermediate splice forms of construct-derived mRNAs. The CYS1-ex1DNR^MUT^-ex2 construct, by contrast, generated a collection of amplicons lacking the PCR fragment corresponding to the normal, WT splice product (Fig. 6B, C and Supplementary Data). Instead, this construct generated a novel lower MW fragment and a spectrum of abnormal higher MW amplicons. The variant caused a shift to the use of an alternative upstream splice donor site as well as an alternative downstream splice acceptor site (Fig. 6 and Supplementary Data). Higher MW bands corresponding to incorrectly spliced transcripts from the mutation-bearing construct are experimental results consistent with in silico based predictions, and independently support the c.318 + 5G > A variant as deleterious. Furthermore, it is known that mutations of the type identified in the patient can lead to diverse, abnormal splicing products^50^.

## Discussion

In the current study, we demonstrate that expression of the *Cys1* transgene in renal collecting ducts of *Cys1*^*cpk/cpk*^ mice rescues the cystic phenotype and down-regulates expression of *Myc* in vivo at the protein and mRNA levels. We also report the first ARPKD patient with a homozygous *CYS1* variant, a c.318 + 5G > A variant predicted to disrupt splicing, which we validated in an in vitro minigene splicing assay. This variant affects the +5 position of the canonical donor splice site, AG/GURAGU. Pathogenic variants affecting +5 position of the donor splice site have been reported in other genes^51^ including the *OXCT1* gene in succinyl-CoA:3-ketoacid CoA transferase (SCOT) deficiency^52^ and the gene encoding fumarylacetoacetate hydrolase in hereditary tyrosinaemia^53^. Detailed analysis of G-to-A sequence changes at the +5 position have revealed disrupted base pairing between donor splice site of a pre-mRNA and the U1snRNP of the spliceosome leading to decreased efficiency of the splice site recognition and exon skipping that can result in frameshifting and premature protein termination^54^. However, this ex vivo system does not allow us to predict whether the variant leads to structural protein alterations (e.g. truncation) and/or decreased abundance of the *CYS1* gene product in vivo.

The identification of a pathogenic *CYS1* variant in a HRFD patient confirms the importance of the *CYS1* gene product for normal function of human collecting duct cells. While it is surprising that *CYS1* deficiency causing ARPKD has been observed in only one family, it is important to note that this gene is GC-rich, particularly its first exon. Such GC-rich regions can be difficult to amplify and sequence using Sanger methodology^55^ and can be missed in next-generation sequencing because low sequence complexity prevents efficient capture prior to library construction^45^. As described in this report, the *CYS1* exon 1 variant was not detected in our initial TRIO-ES analysis but was revealed by WGS. Our recruitment approach for cystic kidney disease patients (pediatric onset of echogenic kidneys and/or 2 + cysts) yielded a cohort, in which 50/79 (63.3%) subjects had a detectable monogenic cause in a cystic kidney disease gene or phenocopy. Among these 50 patients, 32 (64%) had causative variants detected in a NPHP-renal ciliopathy disease gene, while 2/50 (4%) had a causative variant in *PKHD1*. As WGS comes to be more routinely applied in sequencing of patients’ DNA, we speculate that more ARPKD-associated *CYS1* variants will be identified.

Development of collecting duct cysts in humans with presumed *PKHD1* variants and mice with cystin deficiency suggests a shared pathobiology and possibly similar molecular mechanisms underlying cyst formation. In the *cpk* mouse model, renal cysts initially develop at embryonic day 15.5 (E15.5) and are restricted to proximal tubules^56,57^. As *cpk* mice develop and disease progresses, cysts predominantly affect the distal collecting duct region^56^. Specific expression of cystin-GFP in developing ureteric bud-derived collecting ducts rescued the renal cystic phenotype. Transgenic *cpk* R mice expressing the fusion protein only in AQP2-positive collecting duct cells exhibited survival rates and kidney sizes similar to WT mice. Interestingly, while collecting duct cysts were absent in R mice, these animals did develop proximal tubular cysts suggesting that the initial phase of proximal tubular cystogenesis was not rescued. Proximal tubule cysts have also been observed in human ARPKD fetal specimens between 14 and 26 weeks of gestation, but not in the kidneys of fetuses older than 34 weeks of gestation^40^. These observations suggest a gradual shift of cyst formation from proximal tubules to collecting ducts during early fetal development in both human and mouse ARPKD. Consistent with previously published observations^58^ that the *cpk* mutation does not lead to liver pathology on a B6 strain genetic background, examination of liver sections from *cpk* and R mice did not reveal evidence of hepatic lesions.

Cystin is a cilium-associated protein that localizes to the basal bodies and the ciliary axoneme^18,19,59^. Treatment of *cpk* mice with paclitaxel, which promotes microtubule assembly, prevents renal cyst formation, suggesting that cystin may stabilize microtubule assembly within the ciliary axoneme^60^. The primary cilia of the collecting duct epithelium function as transmitters of mechano- and chemosensory stimuli to signaling pathways that regulate multiple key cellular processes including differentiation, proliferation, apoptosis, tissue homeostasis and cell polarity^61^. We have previously demonstrated that cystin, with two functional nuclear localization signals, can be released from the ciliary membrane through a myristoyl-electrostatic switch and translocate to the nucleus where it forms a regulatory complex with necdin to modulate *Myc* expression^25^.

The *Myc* proto-oncogene plays a critical role in normal kidney development^62^ and several lines of evidence suggest a central role for dysregulated *Myc* expression in the pathophysiology of polycystic kidney disease. First, overexpression of *Myc* in the kidneys of SBM transgenic mice causes polycystic kidney disease^63^ and renal cystic disease remitted in a subset of SBM mice that underwent spontaneous reversion to normal kidney *Myc* expression^64^. Second, treatment of *cpk* mice with antisense *Myc* oligonucleotides mitigated the cystic phenotype^23^. Third, *Myc* is overexpressed in mouse models of autosomal dominant polycystic kidney disease (ADPKD)^65,66^ and *Myc* expression appears to be tightly regulated by PC1, the product of the *Pkd1* gene^67^. Fourth, pharmacological inhibition of glucogen synthase kinase 3 beta (GSK3beta), which accelerates cyst formation in *cpk* mice, leads to decreased *My*c expression and amelioration of the cystic phenotype^68^. Similarly, *Myc* is down-regulated in *Cys1*^*cpk/cpk*^; *Smad3*^+/−^ mice and these double mutants have a milder phenotype than *cpk* mice^26^. Our findings that complementation of mutant *cpk* with cystin-GFP rescues the cystic phenotype and restores normal *Myc* expression provides further evidence that cystin acts in vivo as a negative regulator of *Myc*.

In summary, we demonstrate that cystin deficiency causes ARPKD in humans and mice, and that targeted renal expression of a cystin-GFP fusion protein prevents cyst formation in the *cpk* mouse, most probably by downregulating *Myc* expression in collecting duct cells. Our identification of the first case of human ARPKD caused by a *CYS1* variant confirms the relevance of the *cpk* mouse as an ARPKD model yielding important insights into molecular mechanisms underlying disease pathobiology.

## Materials and methods

### Animal study approvals

All mouse experiments were approved by the *Institutional Animal Care and Use Committees* of Children’s National Research Institute and the University of Alabama at Birmingham (UAB), and experiments were carried out in accordance with relevant guidelines and regulations. The study was carried out in compliance with ARRIVE guidelines. Knock-in transgenic mice were generated at the University of Alabama at Birmingham (UAB) Transgenic & Genetically Engineered Models Core facility. *Ksp-Cre* mice were obtained from Jackson Laboratory (Bar Harbor, ME). Mouse colonies were maintained in the animal facility at Children’s National Research Institute.

### Antibodies and lectins

Anti-GAPDH antibody was purchased from Cell Signaling Technologies (# 2118). Anti-AQP2 antibody was purchased from Santa Cruz Biotechnologies (# SC9882). Alexa Fluor 488 conjugated anti-GFP antibody was obtained from Life Technologies (# A21311). Polyclonal rabbit anti-cystin antibody (70,053) was generated in our lab and described previously^19^. Rabbit monoclonal anti-c-Myc antibody was purchased from Abcam (# ab32072). Goat anti-rabbit HRP conjugated secondary antibody was purchased from American Qualex Solution Products (# A102PS). Donkey anti-Goat IgG Alexa Fluor 555 was obtained from Life Technologies (# A21432). Lectins LTA-FITC (# W0909) and DBA-Rhodamine (# Y0828) were obtained from Vector Laboratories.

### Vector cloning

*Cys1-GFP* cDNA was amplified from previously described pEGFP-N1^19^. Gateway PCR primers were used to add flanking attB sites. A one-tube Gateway reaction was performed using pDonr221 and pRosa26 Dest^69^. The reaction product was used to transform competent STBL3 cells (Life Technologies # C7373-03) that were plated on Ampicillin and Kanamycin to select for destination and entry clones, respectively. Destination clones were screened by restriction enzyme digest prior to sequencing. The pRosa26 Dest *Cys1-GFP* targeting vector was linearized with *Kpn1* and electroporated into ES cells. G418 resistant colonies were screened by long-range PCR as described^70^ and positive clones were used to produce chimeric founder mice.

### PCR genotyping

PCR conditions are described in Supplementary Table S1.

### Immunoblotting

Kidney tissue was collected, homogenized, and processed for immunoblotting as previously described^25^. For cystin and control western blots, immuno-reactive protein bands were visualized using SuperSignal West Dura chemiluminescent substrate (Thermo Fisher Scientific # 34076) and exposed to film. For c-MYC and control western blots, images were obtained with a ChemiDoc Imaging System (Bio-Rad laboratory, Inc.) Densitometry was analyzed using Image Lab (Bio-Rad laboratory, Inc., Version 6.0).

### Kidney histology

Tissue samples were collected and fixed in 10% formalin (Fisher Scientific # 23-245-684) for 2 days, then stored in 70% ethanol. The samples were dehydrated, paraffin embedded, cut into 5 µm sections and stained with hematoxylin and eosin (H&E) and slide-mounted for examination by the UAB Comparative Pathology Laboratory.

### Immunofluorescence analysis

Tissue samples were collected from 6 week-old mice and processed, using published methods^34^. Immunofluorescence detection and image acquisition were performed using an Olympus FLUOVIEW FV1000 confocal laser scanning microscope configured with both an Argon Laser (488 nm) and a Laser diode (405 nm, 440 nm, and 559 nm). Images were analyzed using Olympus FV10-ASW 3.0 Viewer software.

### Lectin staining

Five µm sections of fixed paraffin embedded kidney tissues (prepared as described for histopathology) were stained with Rhodamine labeled DBA (Vector Laboratory # RL-1032) and Fluorescein labeled LTA (Vector Laboratory # FL-1321)^71^. Immunofluorescence detection, image acquisition, and analysis were performed as described above.

### Research subjects and study approval

Cystic kidney disease cases with pediatric onset of echogenic kidneys and/or 2 + renal cysts were recruited as previously described^72^. We obtained blood samples and pedigrees following informed consent from individuals with cystic kidney disease or their legal guardians. Clinical data were obtained using a standardized questionnaire (http://www.renalgenes.org). Approval for human subjects research was obtained from Institutional Review Boards of the University of Michigan, Boston Children’s Hospital, and local IRB equivalents, and all procedures were carried out in accordance with relevant guidelines and regulations.

### Exome/genome sequencing and variant calling

For subject B783, TRIO-ES and data processing were performed by the Genomics Platform at the Broad Institute of Harvard and MIT (Broad Institute, Cambridge, MA). Exome sequencing (> 250 ng of DNA, at > 2 ng/μl) was performed using Illumina exome capture (38 Mb target). Single nucleotide polymorphisms (SNPs) and insertions/deletions (indels) were jointly called across all samples using the Genome Analysis Toolkit (GATK) HaplotypeCaller. Default filters were applied to SNP and indel calls using the GATK Variant Quality Score Recalibration approach. Lastly, variants were annotated using the Variant Effect Predictor. For additional information, please refer to the Supporting Information Section S1 in the exome aggregation consortium (ExAC) study^73^. The variant call set was uploaded on to Seqr (https://seqr.broadinstitute.org) and analysis of the entire ES output was performed. TRIO-GS and data processing were performed by the Genomics Platform at the Broad Institute of MIT and Harvard. PCR-free preparation of sample DNA (350 ng input at > 2 ng/µl) is accomplished using Illumina HiSeq X Ten v2 chemistry. Libraries are sequenced to a mean target coverage of > 30×. Genome sequencing data was processed through a pipeline based on Picard, using base quality score recalibration and local realignment at known indels. The BWA aligner was used for mapping reads to the human genome build 38. Single Nucleotide Variants (SNVs) and insertions/deletions (indels) are jointly called across all samples using Genome Analysis Toolkit (GATK) HaplotypeCaller package version 3.4. Default filters were applied to SNV and indel calls using the GATK Variant Quality Score Recalibration (VQSR) approach. Annotation was performed using Variant Effect Predictor (VEP). Lastly, the variant call set was uploaded to *seqr* for collaborative analysis between the CMG and investigator.

For the additional 521 individuals with cystic kidney disease, ES was performed by either the Broad Institute as above or alternatively the Yale Center for Genomics as follows. Sequence reads were mapped against the human reference genome (NCBI build 37/hg19) using CLC Genomics Workbench (version 6.5.1) (CLC bio). Genetic location information is according to the February 2009 Human Genome Browser data, hg19 assembly (http://www.genome.ucsc.edu). Downstream processing of aligned BAM files were done using Picard and Samtools^74^, and SNV calling was done using GATK5.

From both platforms, variant calling was performed in line with proposed guidelines^42^, and the following criteria were employed as previously described^43,44^. The variants included were rare in the population with mean allele frequency < 0.1% and with 0 homozygotes in the adult reference genome databases ExAC and gnomAD. Additionally, variants were non-synonymous and/or located within splice-sites. Based on an autosomal homozygous recessive hypothesis, homozygous variants were evaluated. Subsequently, variant severity was classified based on prediction of protein impact (truncating frameshift or nonsense variants, essential or extended splice-site variants, and missense variants). Splice-site variants were assessed by in silico tools MaxEnt, NNSPLICE, HSF, and CADD splice-site variant prediction scores^75–78^. Missense variants were assessed based on SIFT, MutationTaster and PolyPhen 2.0 conservation prediction scores^79–81^ and evolutionary conservation based on manually derived multiple sequence alignments.

### Homozygosity mapping (HM)

Homozygosity mapping was calculated based on exome sequencing data. In brief, aligned BAM files were processed using Picard and SAMtools4 as described^74^. Single nucleotide variant calling was performed using Genome Analysis Tool Kit (GATK)^82^. The resulting VCF files were used to generate homozygosity mapping data and visual outputs using the program Homozygosity Mapper^83^.

### Web resources used

UCSC Genome Browser, http://genome.ucsc.edu.

Ensembl Genome Browser, www.ensembl.org.

gnomAD browser 2.0.3., https://gnomad.broadinstitute.org.

Polyphen2, https://genetics.bwh.harvard.edu/pph2.

Sorting Intolerant From Tolerant (SIFT), http://sift.jcvi.org.

MutationTaster, www.mutationtaster.org.

Combined Annotation Dependent Depletion, https://cadd.gs.washington.edu.

NNSPLICE splice-site variant prediction, www.fruitfly.org/seq_tools/splice.html.

MaxEnt splice prediction, http://hollywood.mit.edu/burgelab/maxent/Xmaxentscan_scoreseq_acc.html.

Human Splice Finder, www.umd.be/HSF/.

### Mini-gene splicing assay

The minigene assay was used as previously described^49^ to confirm the predicted effect of the patient-derived *CYS1* c.318 + 5G > A variant on splicing. Minigene constructs were engineered in the vector pSpliceExpress^84^ (a kind gift of Dr. Stefan Stamm). *CYS1* DNA insert sequences were synthesized and ligated between pSpliceExpress exons A and B as follows (see Fig. 6): 20 bp of *CYS1* exon 1 3’ sequence, including either the WT or the c.318 + 5G > A patient-derived variant splice donor site, replaced 3’ exon A vector sequences, followed by 200 bp of adjacent *CYS1* intron1 sequence, followed by *CYS1* sequence consisting of the entire *CYS1* exon 2 (53 bp) flanked on either side by 200 bp of adjacent *CYS1* intronic sequences. A positive control construct was also generated, consisting of *CYS1* exon 2, flanked on either side by 200 bp of native *CYS1* intron sequences, ligated between vector exons A and B. Plasmid constructs, including the parent pSpliceExpress vector, were prepared using QIAGEN Plasmid Plus Midi Kit (QIAGEN # 12943) and were eluted in water. Plasmids were transfected into immortalized normal human kidney collecting duct (CD-hTERT) cells (a kind gift of Dr. D. Bell)^85^ using Lipofectamine 2000 Transfection Reagent (Thermo Fisher Scientific # 11668019) according to the manufacturer’s instructions. CD-hTERT cells were cultured in DMEM/F-12 medium (Thermo Fisher Scientific # 11330-057) supplemented with 5% heat-inactivated fetal bovine serum (Thermo Fisher Scientific # 10437-028), 1% penicillin/streptomycin (Thermo Fisher Scientific # 15140-122), 1% L-Glutamine 200 mM (100×, Thermo Fisher Scientific # 25030-081), 200 ng/ml dexamethasone (Sigma-Aldrich # D2915), 1% Insulin-Trasferrin-Selenium solution (Thermo Fisher Scientific # 41400-045), and 1.3 ng/ml 3,3′,5-Triiodo-L-thyronine sodium salt (Sigma-Aldrich # T6397). Following plasmid transfection, cells were maintained in culture for 48 h. Total RNA was then extracted from the cells using RNeasy Mini Kit (QIAGEN # 74104) according to the manufacturer’s instructions. Following treatment with RQ1 RNase-Free DNase (Promega # M6101) for 30 min., the total RNA was re-purified using the RNeasy Mini Kit. cDNA was generated using SuperScript III First-Strand Synthesis SuperMix (Thermo Fisher Scientific # 18080-400) according to manufacturer’s instructions. cDNAs generated from transfected plasmid constructs were amplified by PCR using GoTaq Master Mixes (Promega # M7123) and the primers “Fwd” (5′-CCTGCTCATCCTCTGGGAGC-3′) and “Rev” (5′-AGGTCTGAAGGTCACGGGCC-3′) (see Fig. 6). PCR cycling used an initial denaturation at 95 °C for 2 min, followed by 33 cycles of 95 °C for 30 s, 56 °C for 30 s, 72 °C for 1 min, and final extension at 72 °C for 5 min. The PCR products were resolved by agarose gel electrophoresis. Amplicons of interest (marked by asterisks in Fig. 6) were extracted from agarose gels using QIAquick Gel Extraction Kit (QIAGEN # 28704), TA-cloned with TOPO TA Cloning Kit for Subcloning, and used to transform One Shot TOP10 Chemically Competent *E. coli* (Thermo Fisher Scientific # K450001) according to manufacturer’s instructions. Individual clones were analyzed by restriction digestion and DNA sequencing.

## Supplementary Information

Supplementary Information 1.

Supplementary Information 2.
