# Supplementary material for: Cystin genetic variants cause autosomal recessive polycystic kidney disease associated with altered Myc expression
Source: Sci Rep. 2021 Sep 14;11:18274. doi: 10.1038/s41598-021-97046-4 (PMC8440558; doi:10.1038/s41598-021-97046-4)
Supplement: Supplementary file 1 — Supplementary Information 1. [file 41598_2021_97046_MOESM1_ESM.pdf]

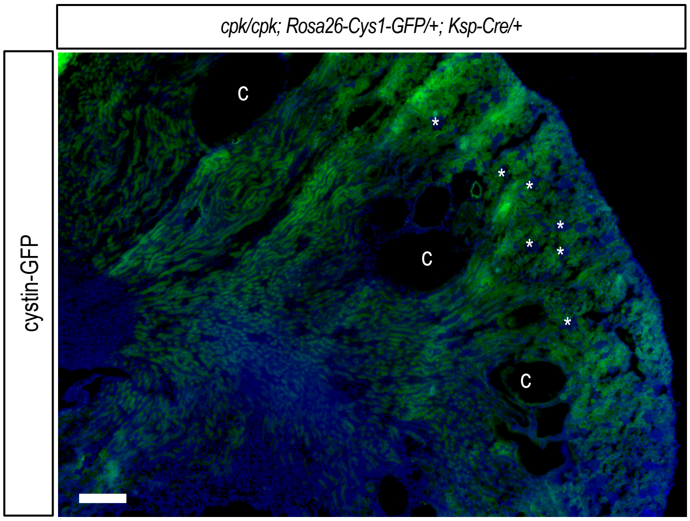

Supplementary Figure S1. Cystin-GFP expression (green) in a 5-week-old rescue mouse kidney was observed in both cortex and medulla. Residual cysts (c) are restricted to the cortico-medullary junction region, consistent with proximal tubule involvement. The asterisks indicate glomeruli (blue) that are entirely GFP negative, indicating that Cystin-GFP expression was mainly in distal tubular elements of the kidney (10X magnification, scale bar equals 100 μm).

Supplementary Figure S2 qRT-PCR analysis showing that kidney *Myc* mRNA levels in Cystin-GFP rescue mice were equivalent to WT. Total RNA was prepared from kidneys of 14-day-old B6 WT, *cpk/cpk; Ksp-Cre/*+ and *cpk/cpk; Rosa26-Cys1-GFP/+; Ksp-Cre/*+ (rescue) mice using RNeasy Mini kit (Qiagen, # 74104), treated with RQ1 RNase-Free DNase (Promega, # M6101) and re-purified using RNeasy Mini kit. RNA samples were reverse transcribed with SuperScript III First-Strand Synthesis SuperMix (Thermo Fisher Scientific, # 18080400) and oligo dT primers. (q)RT-PCR was performed on a QuantStudio 7 Flex Real-Time PCR System (Thermo Fisher Scientific) running the default program, Power SYBR Green PCR Master Mix (Thermo Fisher Scientific, # 4368706), and in c-Myc specific primers 5’- GCC CCC AAG GTA GTG ATC CT -3’ and 5’- GTG CTC GTC TGC TTG AAT GG -3’. *Peptidylpropyl isomerase A* (*Ppia*) expression was used for normalization (primers 5’- AGC ACT GGA GAG AAA GGA TT -3’ and 5’- ATT ATG GCG TGT AAA GTC ACC A -3’). Results were analyzed using QuantStudio Real-Time PCR Software and the ΔΔCt method. The results are presented as mean ± S.E; N=3 per group. Significance ($p<0.05$) was confirmed by Student’s t-test and One-way ANOVA.

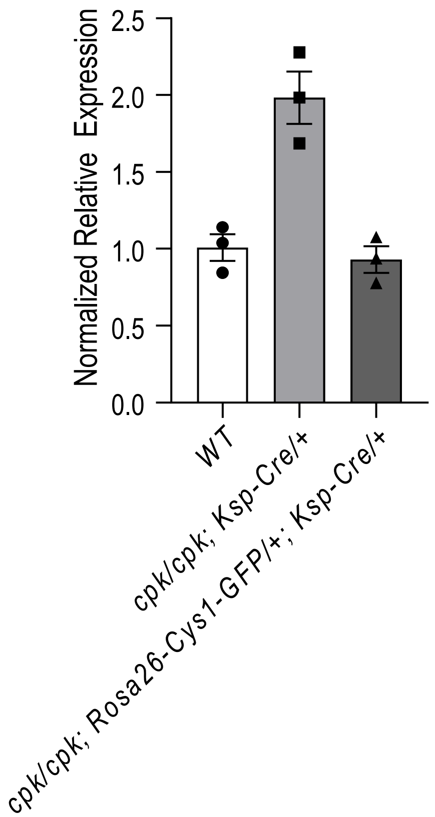

Supplementary Figure S3

A

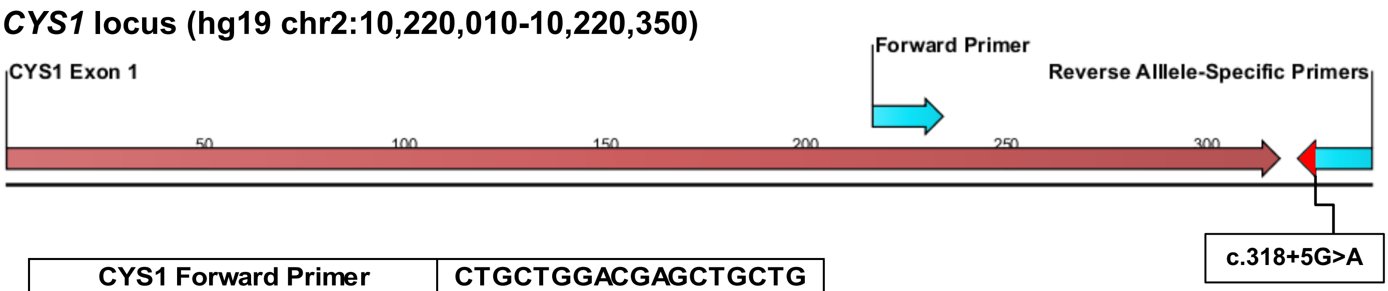

B

CYS1 Forward Primer	CTGCTGGACGAGCTGCTG
CYS1 Reverse WT Primer	GCCCTGCGGCTCCCACAC
CYS1 Reverse MUT Primer	GCCCTGCGGCTCCCACAT

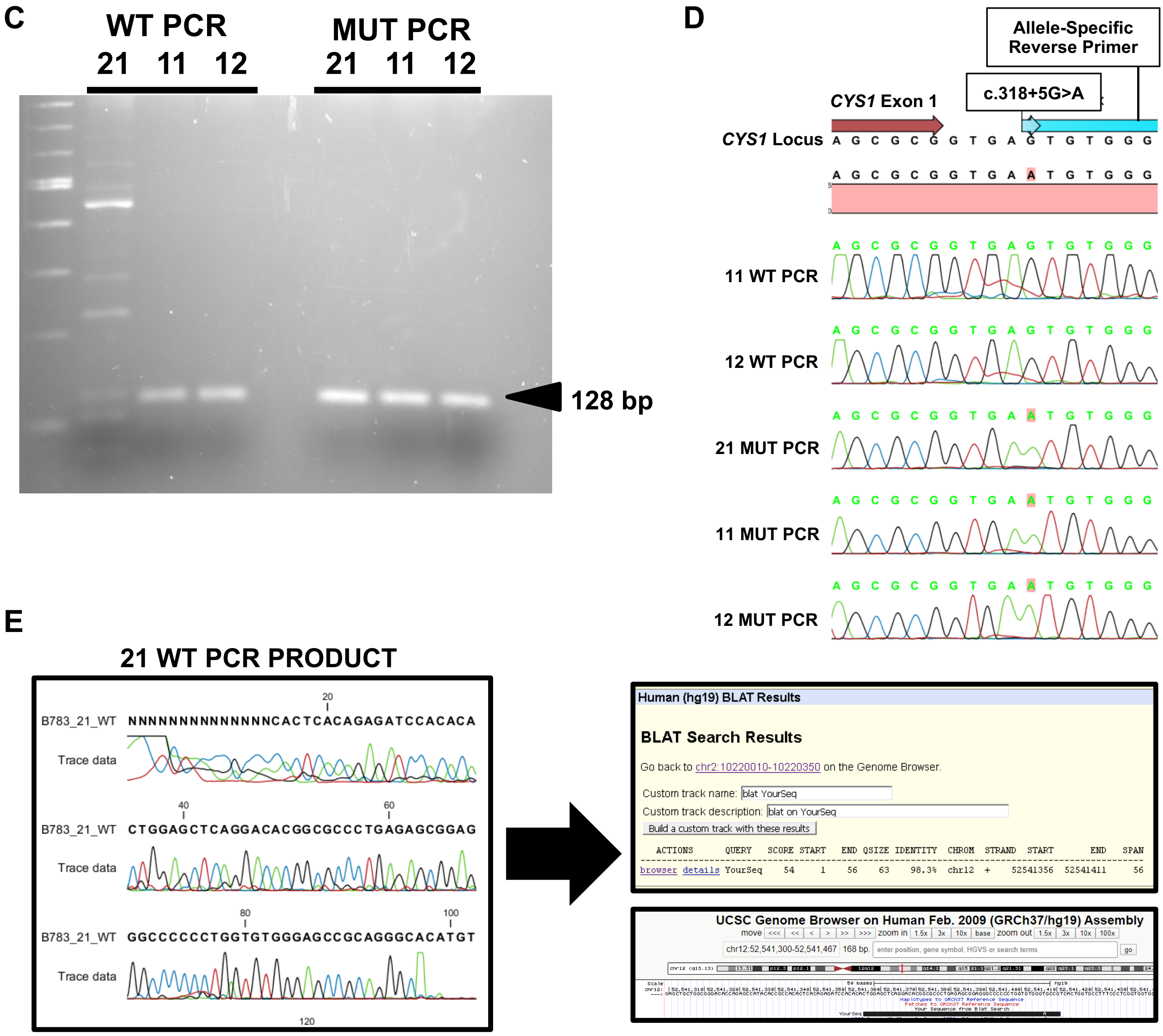

Supplementary Figure S3. Confirmation of *CYS1* variant in family B783 with childhood onset polycystic kidney disease and liver fibrosis. (**A**) Map of *CYS1* locus showing allele-specific primer PCR primer location in context of *CYS1* variant. (**B**) Table shows shared forward primer and allele-specific reverse primers for wildtype (WT) and mutated (MUT) nucleotide. (**C**) Gel electrophoresis of *CYS1* allele-specific PCR products amplified from WT and MUT alleles from proband (21), father (11), and mother (12) DNA in family B783. PCR products of the expected size (128 bp) generated from both parents for WT, while WT PCR primers yielded non-specific bands from the proband. MUT PCR yielded a single product of expected size for all three family members. These results suggest MUT allele heterozygosity for both parents and MUT homozygosity for the proband. (**D**) 128 bp PCR products generated in the 6 reactions in (C) were excised from the gel and Sanger sequenced. The sequences derived from the chromatograms were aligned to the *CYS1* locus as shown. The WT and MUT products from parental DNA (11 and 12) and the MUT product from the affected proband (21) were confirmed as amplification products of *CYS1*. The product from WT PCR of affected proband (21) DNA did not align. (**E**) Sequence chromatogram from proband WT PCR product aligned to the human genome using UCSC Browser BLAT, yielding a single search result indicating 98.3% similarity to an intron in chromosome 12. This finding supports WT PCR generating non-specific products from the proband, while parents yielded *CYS1* products. Abbreviations: 11, father; 12, mother, 21, proband; WT, wildtype; MUT, mutant.

Supplementary Table S1. PCR conditions for mouse genotyping

Transgene	PCR Conditions			Expected Band Sizes	
	Reagents	Primers	Conditions	Wild type	Mutant
Cys1 w/ HotStarTaq PCR[a]	1x PCR buffer, 1.5 mM $MgCl_2$<br>1x Q-Solution<br>50 uM each dNTPs<br>0.4 μM each primer<br>0.025 units HotStarTaq[a] DNA Polymerase<br>4 ng of genomic DNA	F: 5'-TCCTCCCTCCCTATCTCTCCAT-3'<br>R: 5'-ATCCAGCAGGCGTAGGGT-3'	Initial denaturation: 15 min. at 95˚C<br>30 cycles of: 45 sec. at 94˚C<br>1 min. at 57˚C<br>1 min. at 72˚C<br>Final extension: 10 min. at 72˚C	351	315
Ksp-Cre w/ HotStarTaq PCR[a]	1x PCR buffer, 1.5 mM $MgCl_2$<br>1x Q-Solution<br>50 uM each dNTPs<br>0.4 μM each primer<br>0.025 units HotStarTaq[a] DNA Polymerase<br>4 ng of genomic DNA	F: 5'-GCAGATCTGGCTCTCCAAAG-3'<br>R: 5'-AGGCAAATTTTGGTGTACGG-3'	Initial denaturation: 15 min. at 95˚C<br>30 cycles of: 45 sec. at 94˚C<br>1 min. at 55˚C<br>1 min. at 72˚C<br>Final extension: 10 min. at 72˚C	n/a	420
Rosa 26 w/ iProof HF Master Mix[b]	1x iProof HF Master Mix[b]<br>2% DMSO (Bio-Rad)<br>0.4 μM each primer<br>4 ng of genomic DNA	F: 5'-CTCGTGATCTGCAACTCCAG-3'<br>R: 5'-GCTGCATAAAACCCCAGATGACT-3'<br>R: 5'-GCGCATGCTCCAGACTGCCTTG-3'	Initial denaturation: 30 sec. at 98˚C<br>35 cycles of: 30 sec. at 98˚C<br>30 sec. at 61˚C<br>1 min. at 72˚C<br>Final extension: 5 min. at 72˚C	225	319

[a] HotStarTaq PCR (cat#: 203205, QIAGEN)
[b] iProof HF Master Mix (cat#1725310, BIO-RAD)

Supplementary Data

Minigene splicing assay

1. Amplicon analysis*

	Cloned inserts#	Restriction digest&	Successful sequencing**	Result
pSpliceExpress	7/8	5/5	2/2	Both clones match expected sequence
CYS1-ex2	6/8	6/6	2/3	Both clones match expected sequence
CYS1-ex1DNR-ex2	5/8	5/5	1/2	Clone matches expected sequence
CYS1-ex1DNRMUT-ex2	5/8	5/5	2/2	Both clones correspond to use of same alternative donor/acceptor sites

*Amplicon DNA bands were purified from gel (see Fig. 6C) and cloned. #Presence of cloned insert tested by PCR (using Fwd and Rev primers, see Fig. 6); results presented as number positive/total clones tested. &Cloned plasmid DNA analyzed by restriction enzyme digestion. Results presented as number with identical digestion pattern/total number tested. **Multiple clones from each amplicon band underwent sequencing. Results presented as number of successful reads/number of clones sequenced.

2. Amplicon cDNA sequencing results. Primer sequences are underlined. Exon A (vector) is highlighted in green, exon B (vector) is highlighted in blue, *CYS1* exon 1 sequence is highlighted in yellow, and *CYS1* exon 2 is highlighted in purple.

>pSpliceExpress_a1
CTGCTCATCCTCTGGAGCCCCGCCCTGCCCAGGCTTTTGTCAAACAGCACCTTTG
TGGTTCTCACTTGGTGGAAGCTCTCTACCTGGTGTGTGGGGAGCGTGGATTCTTCT
ACACACCCATGTCCCGCCGCGAAGTGGAGGACCCACAAGTGGCACAACTGGAGCT
GGGTGGAGGCCCGTGACCTTCAGACCT
>pSpliceExpress_a2
CTGCTCATCCTCTGGAGCCCCGCCCTGCCCAGGCTTTTGTCAAACAGCACCTTTG
TGGTTCTCACTTGGTGGAAGCTCTCTACCTGGTGTGTGGGGAGCGTGGATTCTTCT
ACACACCCATGTCCCGCCGCGAAGTGGAGGACCCACAAGTGGCACAACTGGAGCT
GGGTGGAGGCCCGTGACCTTCAGACC
>CYS1-ex2_b2
CTGCTCATCCTCTGGAGCCCCGCCCTGCCCAGGCTTTTGTCAAACAGCACCTTTG
TGGTTCTCACTTGGTGGAAGCTCTCTACCTGGTGTGTGGGGAGCGTGGATTCTTCT
ACACACCCATGTCCCGCCGCGAAGTGGAGGACCCCCAAGGTGTGCGCAGAGCAG
AGCACAGAGGGCCACCCGGGGAGCGGCAATGTCTCTGATGGCACAACTGGAGCT
GGGTGGAGGCCCGTGACCTTCAACCT
>CYS1-ex2_b5
CTGCTCATCCTCTGGAGCCCCGCCCTGCCCAGGCTTTTGTCAAACAGCACCTTTG
TGGTTCTCACTTGGTGGAAGCTCTCTACCTGGTGTGTGGGGAGCGTGGATTCTTCT
ACACACCCATGTCCCGCCGCGAAGTGGAGGACCCACAAGGTGTGCGCAGAGCAG
AGCACAGAGGGCCACCCGGGGAGCGGCAATGTCTCTGATGGCACAACTGGAGCT
GGGTGGAGGCCCGTGACCTTCAACCT

>CYS1-ex1DNR-ex2_c7
CTGCTCATCCTCTGGGAGCCCCGCCCTGCCCAGGCTTTTGTCAAACAGCACCTTTG
TGGTTCTCACTTGGTGGAAGCTCTCTACCTGGTGTGTGGGGAGCGTGGATTCTTCT
ACACACCCATGTCCCGCCGCCGCGGTCGCGGGGAGCGCGGTGTGCGCAGAGCAG
AGCACAGAGGGCCACCCGGGGAGCGGCAATGTCTCTGATGGCACAACTGGAGCT
GGGTGGAGGCCCGTGACCTTCAGACC

>CYS1-ex1DNRMUT-ex2_d1
CTGCTCATCCTCTGGGAGCCCCGCCCTGCCCAGGCTTTTGTCAAACAGCACCTTTG
TGGTTCTCACTTGGTGGAAGCTCTCTACCTGGTGTGCGCAGAGCAGAGCACAGAG
GGCCACCCGGGGAGCGGCAATGTCTCTGATGGCACAACTGGAGCTGGGTGGAGG
CCCGTGACCTTCAGACCT

>CYS1-ex1DNRMUT-ex2_d2
CTGCTCATCCTCTGGGAGCCCCGCCCTGCCCAGGCTTTTGTCAAACAGCACCTTTG
TGGTTCTCACTTGGTGGAAGCTCTCTACCTGGTGTGCGCAGAGCAGAGCACAGAG
GGCCACCCGGGGAGCGGCAATGTCTCTGATGGCACAACTGGAGCTGGGTGGAGG
CCCGTGACCTTCAGACCT

3. Schematic illustrating splicing pattern that yielded *CYS1-ex1DNRMUT-ex2* amplicon.

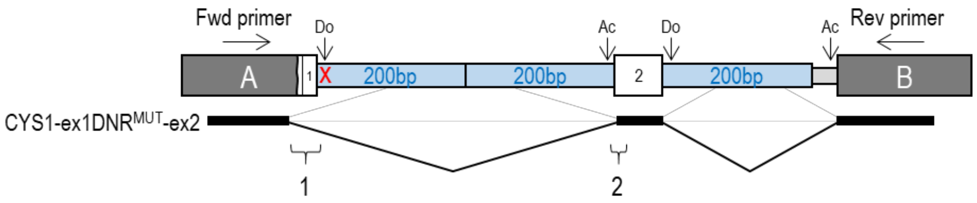

1. Missing 59 bp including CYS1 exon 1 donor site
2. Missing 5 bp of 5' end of CYS1 exon 2

Alignment of CYS1-ex1DNR-ex2_c7 and CYS1-ex1DNRMUT-ex2_d1 & d2 amplicon sequences.

```
                 1                                                  50
CYS1ex1-ex2      CCTGCTCATC CTCTGGGAGC CCCGCCCTGC CCAGGCTTTT GTCAAACAGC
         c7      CCTGCTCATC CTCTGGGAGC CCCGCCCTGC CCAGGCTTTT GTCAAACAGC
         d1      CCTGCTCATC CTCTGGGAGC CCCGCCCTGC CCAGGCTTTT GTCAAACAGC
         d2      CCTGCTCATC CTCTGGGAGC CCCGCCCTGC CCAGGCTTTT GTCAAACAGC

                 51                                      *** ***     100
CYS1ex1-ex2      ACCTTTGTGG TTCTCACTTG GTGGAAGCTC TCTACCTGGT GTGTGGGGAG
         c7      ACCTTTGTGG TTCTCACTTG GTGGAAGCTC TCTACCTGGT GTGTGGGGAG
         d1      ACCTTTGTGG TTCTCACTTG GTGGAAGCTC TCTACCTGGT GTG.......
         d2      ACCTTTGTGG TTCTCACTTG GTGGAAGCTC TCTACCTGGT GTG.......

                 101                                               150
CYS1ex1-ex2      CGTGGATTCT TCTACACACC CATGTCCCGC CGCCGCGGTC GCGGGGAGCG
         c7      CGTGGATTCT TCTACACACC CATGTCCCGC CGCCGCGGTC GCGGGGAGCG
         d1      .......... .......... .......... .......... ..........
         d2      .......... .......... .......... .......... ..........

                 151                                               200
                  ******
CYS1ex1-ex2      CGGTGTGCGC AGAGCAGAGC ACAGAGGGCC ACCCGGGGAG CGGCAATGTC
         c7      CGGTGTGCGC AGAGCAGAGC ACAGAGGGCC ACCCGGGGAG CGGCAATGTC
         d1      .......CGC AGAGCAGAGC ACAGAGGGCC ACCCGGGGAG CGGCAATGTC
         d2      .......CGC AGAGCAGAGC ACAGAGGGCC ACCCGGGGAG CGGCAATGTC

                 201                                              249
CYS1ex1-ex2      TCTGATGGCA CAACTGGAGC TGGGTGGAGG CCCGTGACCT TCAGACCT.
         c7      TCTGATGGCA CAACTGGAGC TGGGTGGAGG CCCGTGACCT TCAGACC..
         d1      TCTGATGGCA CAACTGGAGC TGGGTGGAGG CCCGTGACCT TCAGACCTA
         d2      TCTGATGGCA CAACTGGAGC TGGGTGGAGG CCCGTGACCT TCAGACCT.
```

A 6 nt repeat (GGTGTG, marked with asterisks in the alignment) is present at the sites of alternative splicing in amplicons CYS1-ex1DNRMUT-ex2_d1 & d2. The possible alternative donor-acceptor pairings are therefore TG-TG, GT-GT, TG-GG, GT-CG, or GG-GC.

Corresponds to Figure 1D

IB: Cystin (70053)

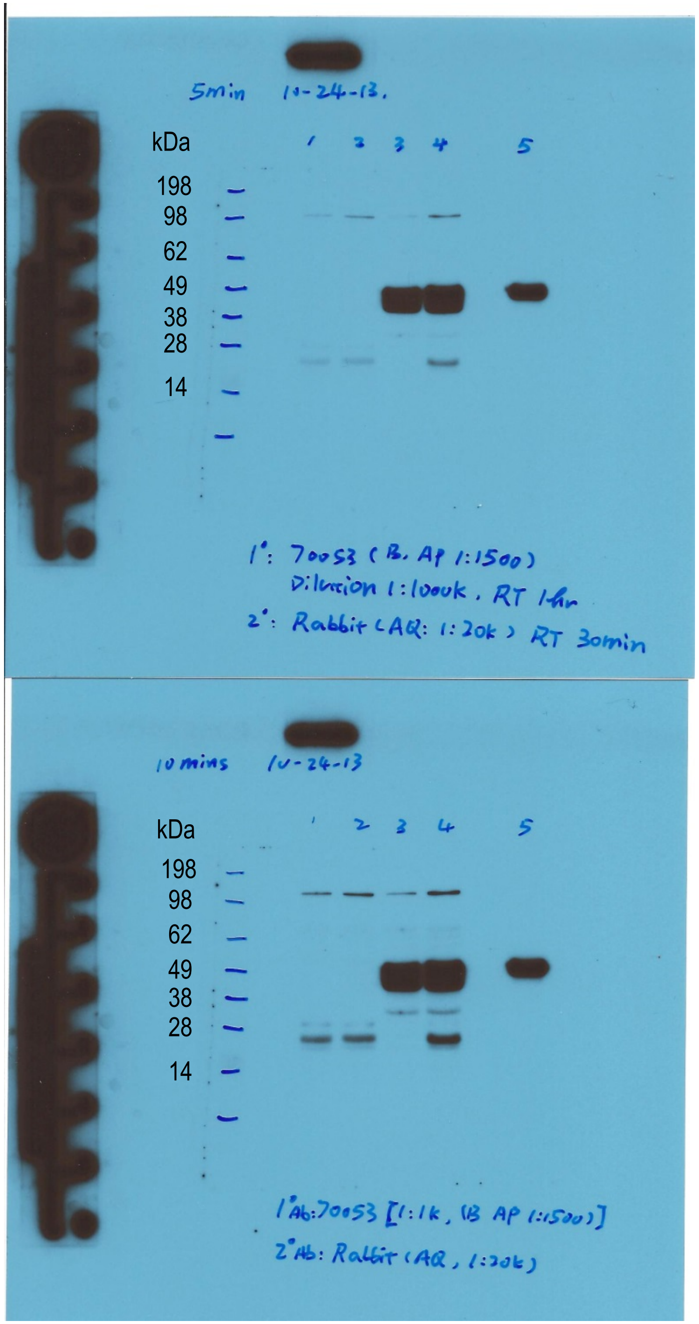

IB: GAPDH

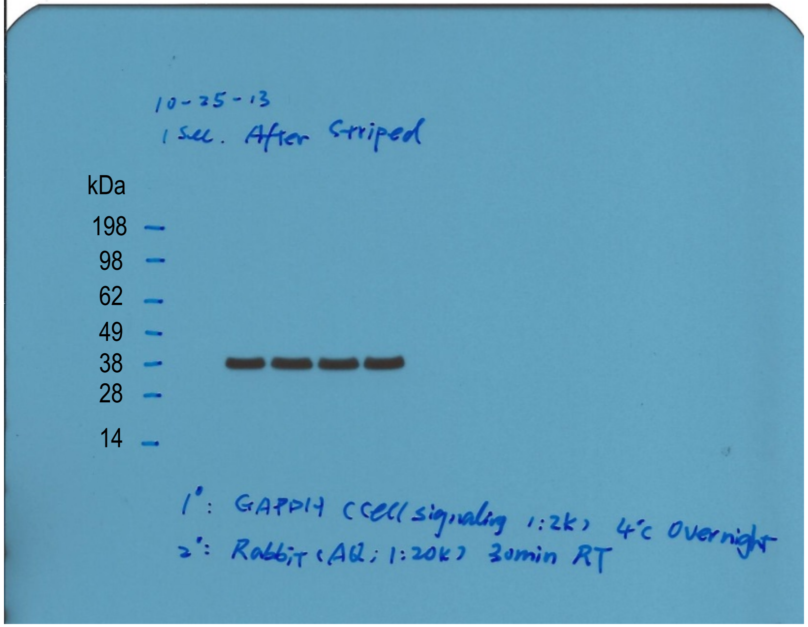

Corresponds to Figure 4A

kDa
198
98
62
49

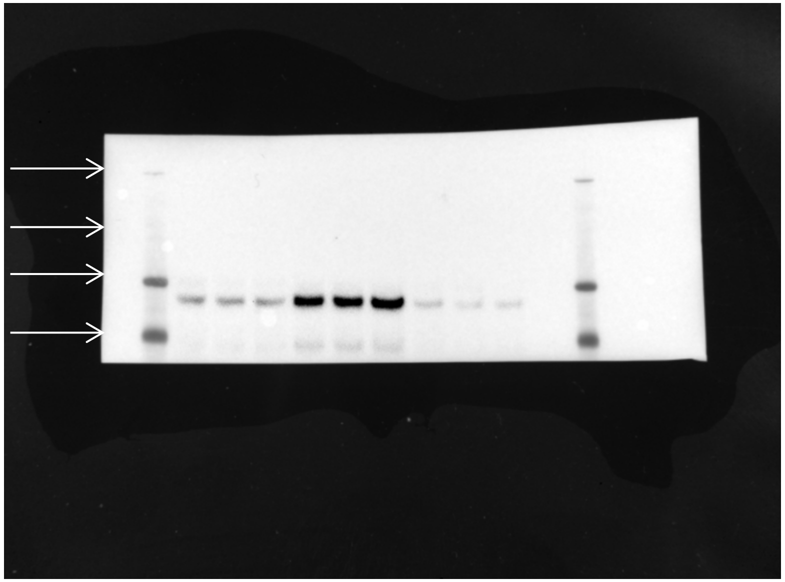

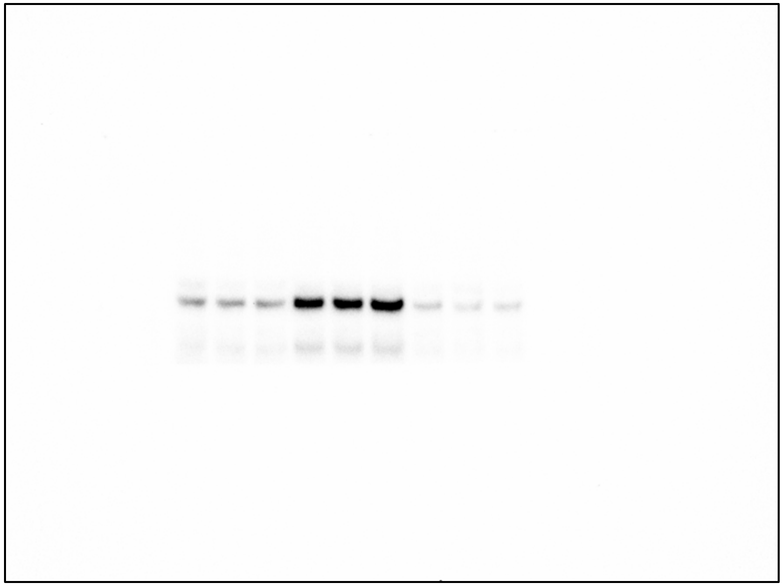

IB: c-Myc

kDa
38
28

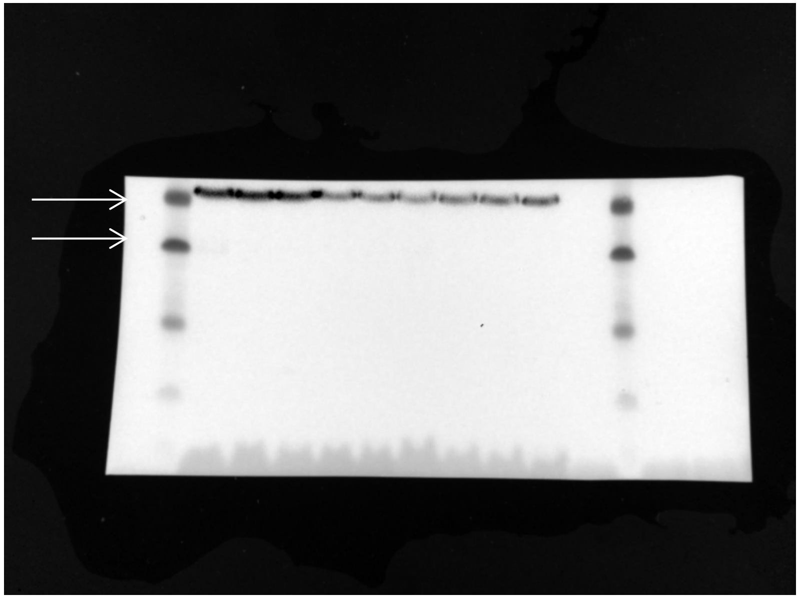

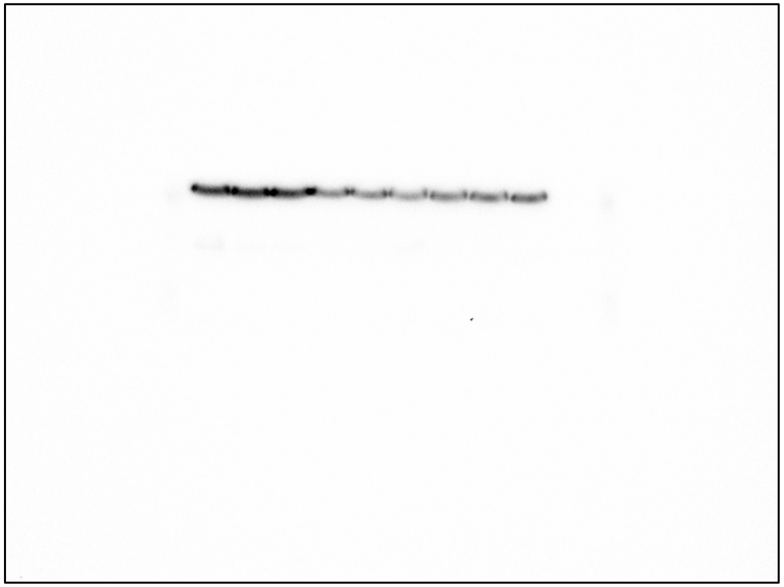

IB: GAPDH
