# Supplementary figures and images for: Cystin genetic variants cause autosomal recessive polycystic kidney disease associated with altered Myc expression
Source: Sci Rep. 2021 Sep 14;11:18274. doi: 10.1038/s41598-021-97046-4 (PMC8440558; doi:10.1038/s41598-021-97046-4)

Corresponds to Figure 1B

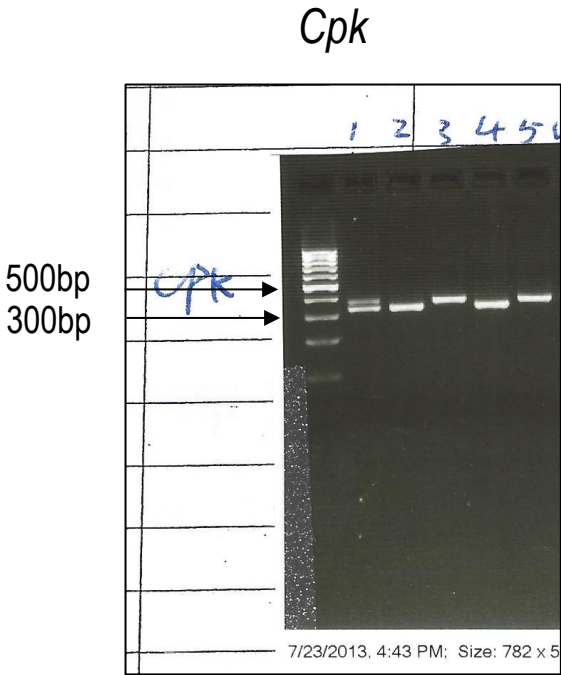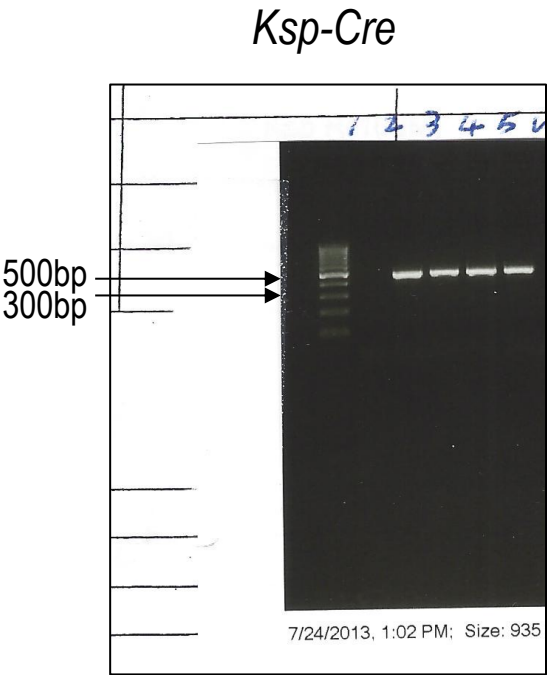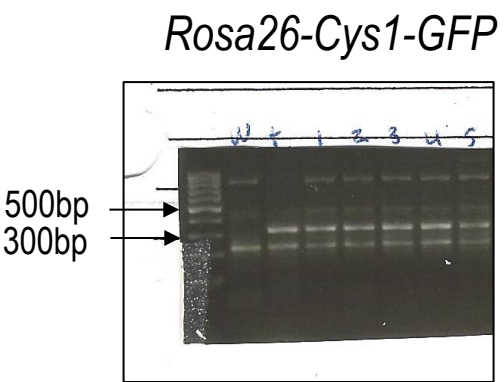

Note: Sample # 1-5 correspond to Lane 1-5 in Figure 1B

Supplement: Supplementary file 2 — Supplementary Information 2. [file 41598_2021_97046_MOESM2_ESM.pdf]
